# The Beneficial Effects of Bisphosphonate-enoxacin on Cortical Bone Mass and Strength in Ovariectomized Rats

**DOI:** 10.3389/fphar.2017.00355

**Published:** 2017-06-07

**Authors:** Xuqiang Liu, Xinhua Qu, Tao Nie, Zanjing Zhai, Haowei Li, Zhengxiao Ouyang, An Qin, Shuhong Zhang, Shuangyan Zhang, Qiming Fan, Tingting Tang, Zhifeng Yu, Min Dai

**Affiliations:** ^1^Department of Orthopedics, Shanghai Key Laboratory of Orthopedic Implant, Shanghai Ninth People’s Hospital, Shanghai Jiaotong University School of MedicineShanghai, China; ^2^Department of Orthopedics, The First Affiliated Hospital of Nanchang University, The Artificial Joint Engineering and Technology Research Center of Jiangxi ProvinceNanchang, China; ^3^Department of Orthopedics, The Second Xiangya Hospital, Central South UniversityChangsha, China

**Keywords:** osteoporosis, bisphosphonate, enoxacin, cortical bone, bone mass, bone strength

## Abstract

Osteoporosis is a major age-related bone disease characterized by low bone mineral density and a high risk of fractures. Bisphosphonates are considered as effective agents treating osteoporosis. However, long-term use of bisphosphonates is associated with some serious side effects, which limits the widespread clinical use of bisphosphonates. Here, we demonstrate a novel type of bone-targeting anti-resorptive agent, bisphosphonate-enoxacin (BE). In this study, ovariectomized rat model was established and treated with PBS, zoledronate (50 μg/kg) and different dose of BE (5 mg/kg and 10 mg/kg), respectively. The rats subjected to sham-operation and PBS treatment were considered as control group. Then, micro-computed tomography scanning, biomechanical tests, nano-indentation test and Raman analysis were used to compare the effects of zoledronate and BE on cortical bone mass, strength, and composition in ovariectomized rats. We found that both zoledronate and BE were beneficial to cortical bone strength. Three-point bending and nano-indentation tests showed that zoledronate- and BE-treated groups had superior general and local biomechanical properties compared to the ovariectomized groups. Interestingly, it seemed that BE-treated group got a better biomechanical property than the zoledronate-treated group. Also, BE-treated group showed significantly increased proteoglycan content compared with the zoledronate-treated group. We hypothesized that the increased bone strength and biomechanical properties was due to altered bone composition after treatment with BE. BE, a new bone-targeting agent, may be considered a more suitable anti-resorptive agent to treat osteoporosis and other bone diseases associated with decreased bone mass.

## Introduction

Bone mass is maintained by osteoblastic bone formation and osteoclastic bone resorption, which is called bone remodeling (Teitelbaum and Ross, 2003; Horne et al., 2005; Karsenty et al., 2009). An imbalance between bone formation and bone resorption causes bone diseases, such as osteoporosis, which has become a worldwide health concern, causing great medical, economic, and social burdens. Two major types of medication are focused on the treatment of osteoporosis, anti-resorptive and anabolic agents (Amugongo et al., 2014). Bisphosphonate (BP), the classical anti-resorptive agent, is effective in preventing the development of osteoporosis and in reducing the risk of fractures (Boivin et al., 2000; Zoehrer et al., 2006; Bala et al., 2011). However, long-term use of BP is associated with some serious side effects (Piesold et al., 2006; Hagen et al., 2014), such as necrosis of the mandibular bone and atypical femoral fracture, which limits the widespread clinical use of BP. Therefore, there is an urgent need to develop a safe, alternative anti-resorptive agent.

Here, we describe a novel type of bone-targeting anti-resorptive agent, bisphosphonate-enoxacin (BE), which functions differently from BP. As previously described, enoxacin, a fluoroquinolone antibiotic, has a great inhibitory effect on osteoclast formation and bone resorption both *in vitro* and *in vivo* (Toro et al., 2012; Liu et al., 2014). It is exciting that a widely used antibiotic has these unexpected properties, which could translate into their clinical use for preventing osteoporosis and other osteoclast-related bone diseases. However, patients may experience adverse effects of the antibiotic, such as dysbacteriosis and gastrointestinal discomfort, with long-term treatment with enoxacin. To overcome these difficulties, we developed a BP derivative of enoxacin, which targets the bone and prevents side effects in other systems. Previous research has shown that BE has a significant inhibitory effect on osteoclast formation *in vitro*, and it is beneficial for preventing orthodontic tooth movement and alveolar bone resorption *in vivo* (Toro et al., 2013; Rivera et al., 2014). However, the effect of BE on bone strength and prevention of bone loss remains unclear. Thus, in the present study, we investigated cortical bone composition and biomechanical properties after treatment with BE in an ovariectomized (OVX) rat model. We hypothesized that the changes in bone mass, bone biomechanical properties, and bone composition would be different in animals treated with BP and BE.

## Materials and Methods

### Animals and Experimental Procedures

An OVX rat model was established as described previously (Peng et al., 2013; Amugongo et al., 2014). Briefly, 60 6-month-old female Sprague–Dawley (SD) rats were ovariectomized (*n* = 48) and sham-operated (*n* = 12). Two months post-surgery, the sham-operated rats were treated with PBS and the ovariectomized rats were randomly divided into four groups and injected intraperitoneally with PBS (vehicle, *n* = 12), zoledronate (ZOL, 50 μg/kg, *n* = 12), low-dose BE (LBE, 5mg/kg, *n* = 12) and high-dose BE (HBE, 10mg/kg, *n* = 12), respectively. Four weeks after the injection, the first cohort of rats (six rats randomly selected from each group) was sacrificed with an overdose of sodium pentobarbital (35 mg/kg; Sigma, St Louis, MO, United States). The right femurs were harvested and placed in 4% formalin for 24 h, then transferred to 70% ethanol for longer-term storage. The left femurs were wrapped in gauze soaked in the saline, and frozen at -20°C until analysis. The remaining rats were injected continuously for another 4 weeks. Then, the second cohort of rats was sacrificed and bone samples were harvested as before. All procedures of this experiment were performed in accordance with the Research Ethics Committee of the Shanghai Ninth People’s Hospital.

### Micro-computed Tomography Scanning

The fixed right femurs were analyzed using high-resolution micro-computed tomography (CT; μCT80, Scanco Medical, Switzerland). A 6-mm region in the middle of the femur was scanned (Amugongo et al., 2014), and the scanning protocol was set at an isometric resolution at 10 μm, and X-ray energy settings of 70 kV and 1170 mA, with a voxel size of 10 μm in all three spatial dimensions. Two hundred consecutive slices at the mid-point of the femur were chosen for further quantitative analysis. The parameters of bone surface/total volume (BS/BV, %), cortical bone thickness (Ct. Th, mm) and percentage of total porosity (%) of each sample were calculated using the software (Image Processing Language, v4.29d, Scanco Medical AG, Bassersdorf, Switzerland) provided with the instrument.(Cheng et al., 2009; Cipriani et al., 2012).

### Biomechanical Testing

The left femurs wrapped in sodium chloride were subjected to biomechanical testing. Samples were placed in 37°C 0.9% sodium chloride for 12 h before testing. About 5 mm of the end of each bone was cut smoothly to decrease the possibility of bucking during the test. Each sample was subjected to a three-point bending test (Model 8874; Instron Corp, Norwood, MA, United States), with a major loading span of 14.5 mm. The bone was loaded to failure at a displacement rate of 0.1 mm/s, and the load and displacement were measured. After testing, the peak load (N) was recorded from the maximum load, and the yield and ultimate strength (Pa) of the femur were calculated automatically. All tests were conducted blindly.

### Nano-indentation Measurement

After micro-CT scanning, the right femurs were subjected to nano-indentation testing. Prior to testing, specimens were thawed to room temperature, ground, and polished as previously reported (Lopez Franco et al., 2011). A Nano Indenter XP system (MTS Nanoindenter XP, Oak Ridge, TN, United States) was employed to measure the force and displacement during the indentation of the polished bone specimen. For each specimen, at least ten indentation points were made on the mid-shaft anterior surface of the femur bone. The measurement areas were determined using an optical microscope at 50 × magnification. A Berkovich shape diamond indenter tip (Ei = 1141 Gpa, vi = 0.07) was used to perform the nano-indentation tests at each site. The indentation procedure was under displacement control. After the surface was identified, the indenter was advanced to 500 nm at a speed of 10 nm/s to avoid effects of bone surface roughness. A typical indentation load-displacement curve includes a loading segment, a 10-s holding period at maximum load, an unloading segment, and a 50-s holding period for thermal drift measurement at 10% of the maximum load elastic modulus. Finally, elastic modulus and hardness, which reflect intrinsic properties of cortical bone was recorded analyzed.

### Raman Analysis

After biomechanical testing, the left femurs were dehydrated in graded concentrations of ethanol prior to Raman analysis. All measurements were obtained with a spatial resolution of ∼1 μm. The laser power (wavelength, 785 nm) for all measurements was 100 mW. The Raman spectra were obtained with an acquisition time of 5 s applying 10 co-additions. The following Raman parameters were calculated, as published elsewhere: (1) the relative proteoglycan content was expressed as the proteoglycan/organic matrix ratio (the ratio of the integrated areas of the proteoglycan/CH3 [1365 to 1390 cm^-1^] band [representative of glycosaminoglycans to the Amide III band]) (Koljenovic et al., 2004; Ellis et al., 2009); and (2) the maturity/crystallinity of the bone mineral apatite crystallites was approximated based on the full-width at half-height (FWHH) of the v_1_PO_4_ (930 to 980 cm^-1^) band, which has been shown to correlate inversely with crystallite length (Morris and Mandair, 2011; Thompson et al., 2011; Yamamoto et al., 2012). Two different parameters, proteoglycan content and mineral maturity/crystallinity, were calculated. Proteoglycans have been proposed to prevent the mineralization of osteocyte canaliculi (Irie et al., 2008; Thompson et al., 2011), and mineral maturity/crystallinity was considered to be associated with mechanical properties (Fratzl et al., 1994; Jager and Fratzl, 2000).

### Statistical Analysis

The data were expressed as mean ± SD and analyzed by one-way analysis of variance (ANOVA). The Student *t*-test was conducted for the comparisons between individual groups using SPSS 13.0 software (SPSS Inc., United States). *P* < 0.05 indicated a significant difference between groups.

## Results

### Effect of BE on Cortical Bone Microarchitecture

**FIGURE 1:**
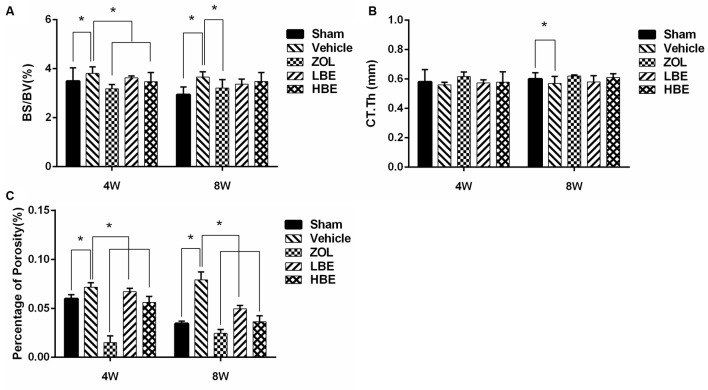
Effect of BE and zoledronate on cortical bone microarchitecture. The middle of the right femurs from OVX rats was subjected to micro-computed tomography scanning and parameters were analyzed. Bone surface/total volume **(A)** and percentage of porosity **(C)** obviously increased in the OVX groups. After treatment with zoledronate and BE, a significant decrease was observed. Compared with the sham groups, cortical bone thickness **(B)** reduced in the OVX groups, but a significant difference was only observed in the 8-week treatment group. After treatment with zoledronate and BE, a non-significant trend was observed for improvement in cortical bone thickness. ^∗^*P* < 0.05.

Our results showed that cortical bone thickness was significantly lower in the OVX groups compared to the sham groups. However, both zoledronate and BE improved cortical bone thickness after treatment. Additionally, zoledronate-treated groups showed superior cortical bone thickness compared to the groups treated with BE, though there was no significant difference between them (**Figure 1B**). BS/BV and the percentage of total porosity were significantly higher in the OVX groups than in any other group. After treatment with zoledronate and two different concentrations of BE for 4 weeks, BS/BV and the percentage of total porosity decreased and there was a significant difference between the OVX group and the zoledronate and high-dose BE groups. Additionally, a much lower BS/BV and percentage of total porosity was seen after treatment of zoledronate and BE for 8 weeks (**Figures 1A,C**).

### Effect of BE on Cortical Bone Mechanical Properties

**FIGURE 2:**
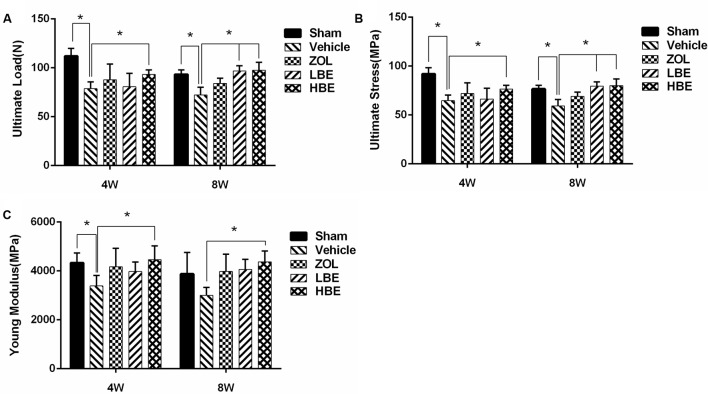
Effect of BE and zoledronate on biomechanical properties. The three-bending test was used, and three parameters were analyzed: ultimate load **(A)**, ultimate stress **(B)**, and Young modulus **(C)**. Cortical bone biomechanical properties were ameliorated after treatment with BE and zoledronate, with superior properties observed in the high-dose BE group. ^∗^*P* < 0.05.

As shown in **Figure 2**, the ultimate load was apparently decreased in the OVX groups, but when treated with zoledronate or different concentrations of BE for 4 or 8 weeks, it significantly improved. Ultimate load was greater in BE-treated groups than in the zoledronate-treated groups. Compared to the group treated with BE for 4 weeks, the 8-week-treated group showed a higher ultimate load (**Figure 2A**). Both hardness and Young modulus were obviously improved after treatment with BE. There was a significant difference between the OVX group and the BE-treated groups (**Figures 2B,C**). Moreover, the effect of BE on cortical bone mechanical properties showed a dose-dependent trend. Compared to the group treated with low concentration of BE, superior biomechanical properties were observed in the high concentration of BE group.

**FIGURE 3:**
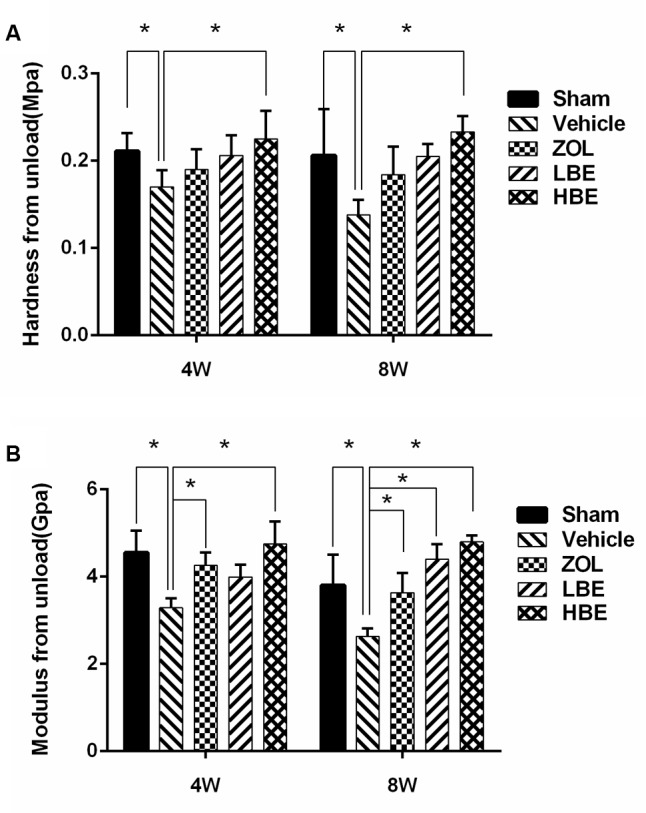
Tissue-level biomechanical properties were investigated using the nano-indentation test. Cortical bone hardness **(A)** and elastic modulus **(B)** improved after treatment with BE and zoledronate for 4 weeks, especially in the high-dose BE group. ^∗^*P* < 0.05.

To determine whether BE injection affects tissue-level biomechanical properties, nano-indentation testing was performed (**Figure 3**). Elastic modulus and hardness were significantly increased following treatment with BE compared to the OVX groups. Additionally, both the low- and high-dose BE groups showed superior mechanical properties to those in the group treated with zoledronate. When comparing the effect of the 4- and 8-week-treated groups, elastic modulus and hardness in the 8-week group were higher than that in the 4-week group, but not significantly so.

### Effect of BE on Composition of Cortical Bone

It is known that the degree of bone matrix mineralization is the key factor in the determination of bone strength (Roschger et al., 2008; Gamsjaeger et al., 2010). In accordance with previous research (Gamsjaeger et al., 2010), we used Raman analysis to investigate bone composition. The peak intensity of phosphate band (approximately 959 cm^-1^) was obviously increased in both zoledronate- and BE-treated groups, indicating that inorganic substance was improved in the bone. Interestingly, comparing to OVX groups, AmideIII band (1243–1230 cm^-1^), which reflects the content of bone organic matrix, was increased in both two different concentrations of BE treated groups. However, with the treatment of zoledronate for 4 and 8 weeks, the intensity of AmideIII band was not superior to the OVX groups. So, we hypothesized that the content of bone organic matrix was not increased following the improvement of inorganic substance after the treatment of zoledronate. To prove the hypotheses, further analysis was conducted as we described in materials and methods, and our results showed that both organic and inorganic matrix significantly decreased when the rats were subjected to OVX operation (**Table 1**). With the treatment of zoledronate, the content of the inorganic matrix was obviously improved. However, the proteoglycan content normalized to the amount of organic matrix was not associated with the increase of inorganic matrix. Both organic and inorganic matrix was significantly higher in the BE-treated groups than in the OVX group, and compared with the zoledronate-treated group, the organic matrix was apparently improved in the BE-treated groups. Additionally, after BE treatment for 8 weeks, the ratio of organic matrix to inorganic matrix was similar to that in the sham group.

**Table 1:** Summary table for all the results.

	4 Weeks					8 Weeks				
	
	Sham	Vehicle	ZOL	LBE	HBE	Sham	Vehicle	ZOL	LBE	HBE
**Bone composition parameters by Raman analysis**				
Proteoglycans content (%)	3.0 ± 0.4	1.8 ± 0.2*	1.9 ± 0.15	2.9 ± 0.31#	3.1 ± 0.26#	2.9 ± 0.35	1.87 ± 0.38*	2.0 ± 0.40	2.7 ± 0.17#	3.1 ± 0.3#
Mineral maturity	23.4 ± 0.53	22.6 ± 0.2*	22.6 ± 0.42	22.7 ± 0.56	22.9 ± 0.2	23.9 ± 0.4	23.0 ± 0.35	23.1 ± 0.35	23.3 ± 0.15	23.6 ± 0.13
**Cortical bone microarchitecture by Micro-CT**				
BS/BV (%)	3.49 ± 0.54	3.81 ± 0.27*	3.18 ± 0.17#	3.63 ± 0.07	3.47 ± 0.38#	2.94 ± 0.31	3.66 ± 0.21*	3.21 ± 0.35#	3.37 ± 0.21	3.48 ± 0.36
Ct.Th (mm)	0.58 ± 0.085	0.56 ± 0.017	0.616 ± 0.032	0.574 ± 0.02	0.579 ± 0.07	0.6 ± 0.042	0.57 ± 0.047*	0.62 ± 0.007	0.58 ± 0.043	0.61 ± 0.026
Percentage of porosity (%)	0.0597 ± 0.0042	0.0715 ± 0.0047*	0.015 ± 0.007#	0.067 ± 0.0034#	0.056 ± 0.006#	0.0345 ± 0.0023	0.079 ± 0.0082*	0.0245 ± 0.004#	0.0495 ± 0.0034#	0.0363 ± 0.006#
**Cortical bone mechanical properties by Biomechanical testing**				
Ultimate load (N)	111.94 ± 7.87	78.69 ± 6.84*	87.69 ± 16.27	80.7 ± 13.6	93.18 ± 4.8#	93.33 ± 4.57	72.19 ± 8.03*	84.06 ± 5.19	96.76 ± 5.38#	97.51 ± 8.1#
Ultimate stress (MPa)	91.96 ± 6.47	64.65 ± 5.62*	72.04 ± 10.79	66.3 ± 11.17	76.55 ± 3.92#	76.67 ± 3.76	59.3 ± 6.59*	69.06 ± 4.26	79.49 ± 4.42#	80.11 ± 6.66#
Young modulus (MPa)	4332.0 ± 401.7	3386.3 ± 433.3*	4174.1 ± 749.8	3976.9 ± 390.5	4455.2 ± 572.3#	3879.5 ± 871.5	2998.6 ± 323.2	3976.5 ± 713.4	4056.6 ± 416.6	4372.8 ± 440.1#
**Cortical bone mechanical properties by Nano-indentation measurement**				
Hardness from unload	0.211 ± 0.0205	0.17 ± 0.019*	0.19 ± 0.023	0.206 ± 0.023	0.225 ± 0.032#	0.206 ± 0.053	0.138 ± 0.017*	0.184 ± 0.032	0.205 ± 0.014	0.233 ± 0.018#
Modulus from unload	4.554 ± 0.498	3.286 ± 0.222*	4.257 ± 0.303#	3.994 ± 0.283	4.75 ± 0.516#	3.803 ± 0.698	2.626 ± 0.186*	3.635 ± 0.453#	4.4 ± 0.344#	4.796 ± 0.154#


*^∗^*P* < 0.05 compared to the Sham group; ^#^*P* < 0.05 compared to the Vehicle group.*

## Discussion

In this study, we described a new agent, BE, which targets the skeletal system. As in previous research by Toro et al., BE showed a great inhibitory effect on osteoclast formation and bone resorption both *in vitro* and *in vivo* (Toro et al., 2013). However, the effect of BE on bone strength and prevention of bone loss remains unclear. Thus, we evaluated BE in an OVX rat model. Attention was focused on the influence of BE and zoledronate on cortical bone mass and strength.

Micro-CT scanning demonstrated that bone loss occurred quickly in OVX rats, resulting in a significant decrease in cortical bone thickness and an obvious increase of cortical bone surface and percentage of porosity. However, treatment with zoledronate and BE for 4 and 8 weeks reduced cortical bone thickness and increased bone surface, and the percentage of porosity was rescued. Thus, both BE and zoledronate were beneficial for preventing bone loss, and there was no significant difference between them in improving cortical bone thickness and reducing the percentage of porosity. Additionally, both BE- and zoledronate-treated groups showed superior mechanical properties to those in the OVX groups. Unexpectedly, femurs from the BE-treated groups (i.e., the low- and high-dose groups) were harder and had a higher modulus than those from zoledronate group. Therefore, we hypothesized that differences in bone mechanical properties in the BE and zoledronate groups may have been due to alterations of bone composition. The Raman measurement was used to analyze cortical bone composition. We showed that the BE-treated OVX rats had a higher proteoglycan content and better mineral maturity compared with the rats treated with zoledronate, which may explain the higher cortical bone strength observed in rats treated with BE than in those treated with zoledronate, although further investigation is needed to confirm this hypothesis.

Bone matrix is primarily composed of hydroxyapatite crystallites (inorganic matrix) and collagen fibrils (organic matrix), which contribute to excellent bone mechanical performance due to high stiffness and toughness; this is accomplished by a synergy between matrix organization and composition (Kazanci et al., 2006, 2007). Changes in bone, especially cortical bone composition, will obviously affect its biomechanical properties. It is known that osteoporosis is a major age-related bone disease characterized by low mineralization. With reduced bone matrix mineralization, patients with osteoporosis usually experience increased bone fragility and low bone strength (Roschger et al., 2008). BPs were commonly used in clinics as anti-resorptive agents. They also reduce cortical bone porosity (Roschger et al., 2001; Bala et al., 2014; Chavassieux et al., 2014; Misof et al., 2014), mildly increase cortical bone thickness (Seeman et al., 2010), and improve cortical bone strength (Gasser et al., 2008). However, long-term use of BP is associated with some serious side effects, as noted above.

BE, a BP derivative of enoxacin, may be a safe, alternative anti-resorptive agent. It has the same effects as zoledronate on preventing cortical bone loss, but it is superior to zoledronate in enhancing cortical bone strength by altering and rationalizing the composition of bone materials. BE’s novel properties and satisfactory therapeutic effects suggest that BE has great potential to be developed as a novel bone-targeting anti-resorptive agent to be used in the treatment of osteoclast-related diseases.

## Ethics Statement

This study was carried out in accordance with the recommendations of ‘Guidance for Animal Research, Ethical Committees for Animal Research of Shanghai Ninth People’s Hospital Affiliated to Shanghai Jiao Tong University, School of Medicine.’ The protocol was approved by the Ethical Committees for Animal Research of Shanghai Ninth People’s Hospital Affiliated to Shanghai Jiao Tong University, School of Medicine (Protocol Number: HKDL[2013]50).

## Author Contributions

XL, XQ, TN, ZY, and MD designed the study. XL and XQ wrote the paper. XL, XQ, TN, ZZ, HL, ZO, ZY, and MD modified the paper. XL, XQ, TN, ZZ, HL, and ZO performed the experiments. XL and XQ conducted statistical analysis. All authors reviewed the results, made substantial contributions and approved the final version of the manuscript.

## Conflict of Interest Statement

The authors declare that the research was conducted in the absence of any commercial or financial relationships that could be construed as a potential conflict of interest.
